# The kinetic glomerular filtration rate is not interchangeable with measured creatinine clearance for prediction of piperacillin underexposure in critically ill patients with augmented renal clearance

**DOI:** 10.1186/s13054-018-2117-7

**Published:** 2018-07-23

**Authors:** Cédric Carrié, Sébastien Rubin, Pierre Sioniac, Dominique Breilh, Matthieu Biais

**Affiliations:** 1 Anesthesiology and Critical Care Department, CHU Bordeaux, 33000 Bordeaux, France; 2 Nephrology Department, CHU Bordeaux, 33000 Bordeaux, France; 3 Pharmacy and Clinical Pharmacy Department, CHU Bordeaux, 33000 Bordeaux, France; 4 Pharmacokinetics and PK/PD Group INSERM 1034, University Bordeaux, 33000 Bordeaux, France; 5 University Bordeaux Segalen, 33000 Bordeaux, France; 6 Surgical and Trauma Intensive Care Unit, Anesthesiology and Critical Care Department, Hôpital Pellegrin, CHU Bordeaux, Place Amélie Raba Léon, 33076 Bordeaux Cedex, France

**Keywords:** Augmented renal clearance, Kinetic glomerular filtration rate, Piperacillin, Critical care

In the critical care setting, augmented renal clearance (ARC) is increasingly recognized as one of the leading causes of subtherapeutic antibiotic exposure [1]. However, commonly used formulas for estimating glomerular filtration rate (GFR) are inaccurate in patients with ARC and the 24-h urinary creatinine clearance (Cr_CL_) remains the best available approach for optimizing empirical antimicrobial therapy [2]. On the other hand, no study has evaluated the clinical and prognostic value of the kinetic estimated GFR (KeGFR) in this context. We thus aimed to determine whether KeGFR could be a reliable alternative to measured Cr_CL_ in critically ill patients needing early initiation of an appropriate piperacillin dosing regimen.

For this purpose, we retrospectively analyzed 60 consecutive patients who underwent 24-h urinary Cr_CL_ measurements and therapeutic drug monitoring during the first 3 days of antimicrobial therapy of piperacillin administered 16 g/day continuously. The protocol pertaining to this substudy has been published elsewhere [3]. As previously described, the corresponding KeGFR was calculated as follows: $\frac{\mathrm{Baseline}\ \mathrm{sCr}\ \mathrm{x}\ \mathrm{eGFR}}{\mathrm{Mean}\ \mathrm{sCr}}\times \left(1-\frac{24\ \mathrm{x}\ \Delta \mathrm{sCr}}{\Delta \mathrm{t}\ \mathrm{x}\ \mathrm{Max}\Delta \mathrm{sCr}/\mathrm{Day}}\right)$ with eGFR derived from the CKD-EPI (Chronic Kidney Disease Epidemiology Collaboration) equation using serum creatinine (sCr) before admission, Δt fixed at 24 h between two sCr measurements, and maximal sCr increase per day approximated to 133 μmol/L [4]. ARC was defined by a measured Cr_CL_ ≥ 130 mL/min/1.73 m^2^. Piperacillin underdosing was arbitrarily defined by a free drug concentration ≤ 32 μg/ml at steady state.

Among the 180 samples analyzed, the incidence of ARC was 48% (median Cr_CL_ values = 124 [83–170] ml/min/1.73 m^2^) and the incidence of piperacillin underdosing was 51% (median piperacillin concentrations = 32 [22–47] μg/ml). The diagnostic agreement between KeGFR and Cr_CL_ was only moderate (κ = 0.48 [95% confidence interval 0.4–0.55]) (Fig. 1). Comparison between KeGFR and Cr_CL_ showed a mean bias of − 8.7 ml/min/1.73 m^2^ and limit of agreement from − 99 ml/min/1.73 m^2^ to 82 ml/min/1.73 m^2^. Finally, the area under the ROC curve generated for KeGFR was significantly lower than the one generated for measured Cr_CL_ for prediction of piperacillin underdosing (0.76 [0.68–0.83] vs 0.85 [0.79–0.91], *p* = 0.03; Fig. 2).

**Fig. 1:**
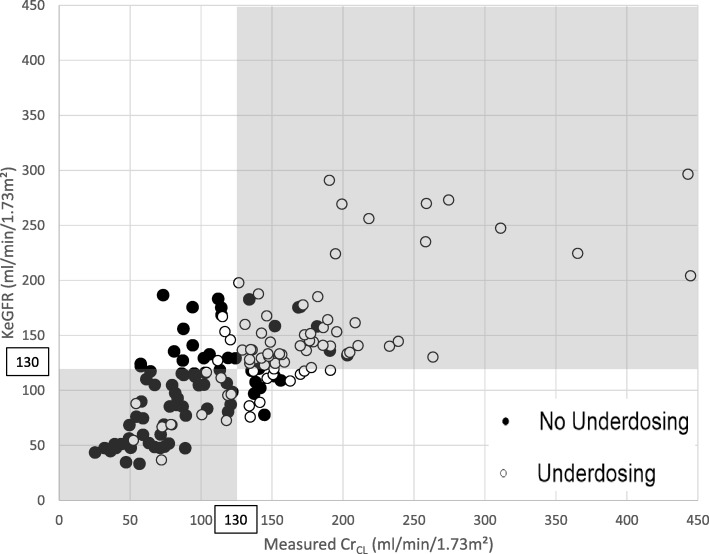
Correlation between measured Cr_CL_ and KeGFR (r^2^ = 0.54, *p* < 0.0001) and repartition of samples with (*white circles*) or without (*black circles*) piperacillin underdosing, defined by an unbound concentration ≤ 32 μg/ml. ARC was defined by a measured Cr_CL_ or a KeGFR ≥ 130 ml/min/1.73 m^2^. Samples in the *gray shaded area* are considered to be well classified

**Fig. 2:**
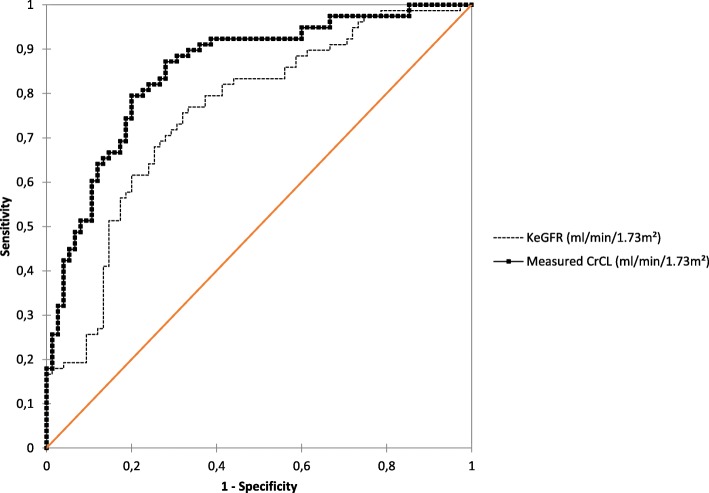
Receiver operating characteristics (ROC) curves evaluating the ability of KeGFR and measured Cr_CL_ to predict piperacillin underdosing. Areas under ROC curves between KeGFR and measured Cr_CL_ were compared using the Handley approach. Piperacillin underdosing was defined by a free drug concentration ≤ 32 μg/ml

In conclusion, KeGFR is not interchangeable with measured Cr_CL_ for prediction of piperacillin underexposure in critically ill patients with ARC. Also, scarce data may suggest a better predictive value of Cockcroft-Gault compared to MDRD (Modification of Diet in Renal Disease Study) or CKD-EPI for identifying patients with ARC [5]; a measured CL_CR_ should be performed to accurately guide drug dosing. This study emphasizes the need for dosing adjustment and therapeutic drug monitoring in patients with ARC.

## Abbreviations

ARC: Augmented renal clearance
Cr_CL_: Creatinine clearance
GFR: Glomerular filtration rate
KeGFR: Kinetic estimated glomerular filtration rate
MIC: Minimum inhibitory concentration
ROC: Receiver operating characteristic
sCr: Serum creatinine
